# Additional dydrogesterone for the treatment of chronic endometritis treated with antibiotic in premenopausal women with endometrial polyps: a retrospective cohort study

**DOI:** 10.1186/s12905-022-02033-0

**Published:** 2022-11-05

**Authors:** Yue Liu, Xin Yu, Jing Huang, Chengchao Du, Honggui Zhou, Yamei Yang, Dacheng Qu

**Affiliations:** 1 Department of Obstetrics and Gynecology, Affiliated Hospital of North Sichuan Medical College, No 63, Wenhua Road, Nanchong, China; 2 Non-invasive and Microinvasive Laboratory of Gynecology, Affiliated Hospital of North Sichuan Medical College, 637000 Nanchong, China; 3 Department of Obstetrics and Gynecology, LongQuanYi District of maternity and child health care hospital, Chengdu, China

**Keywords:** Antibiotic, Chronic endometritis, Combination, Dydrogesterone, Endometrial scratching

## Abstract

**Background:**

To assess the efficacy of dysdrogesterone in the treatment of chronic endometritis (CE) treated with antibiotic in premenopausal women with endometrial polyps (EPs).

**Methods:**

Routine detection of endometrium was simultaneously conducted to determine whether there was CE by syndecan-1 (CD138), while women underwent hysteroscopic polypectomy in our hospital. Antibiotic was given for the treatment of CE. A total of 235 premenopausal women with CE who underwent hysteroscopic polypectomy were enrolled in the retrospective observational study. In the control group, single antibiotic was given for the treatment of CE form January 2016 to December 2018, and in the treatment group additional dydrogesterone was used from January 2019 to November 2020. Comparison of cure rates of CE with different treatment regimens was performed.

**Results:**

The cure rates of CE in dydrogesterone and antibiotic combination group and the single antibiotic group were 85.2% and 74.3%, respectively, with overall cure rate of 80.0% (188/235). The combination group showed better effects regarding the cure rate of CE (P < .05). Multivariate analysis confirmed that the cure rate of CE was not affected by age, body mass index, number of EPs, the status of estrogen receptor and the status of progesterone receptor. Conversely, dydrogesterone and endometrial scratching were beneficial factors for cure rate increase with antibiotic treatment.

**Conclusion:**

Combination of dydrogesterone and antibiotic was more effective for cure rate of CE than antibiotic alone in premenopausal women after hysteroscopic polypectomy. Endometrial scratching also contributed to the cure rate increase with antibiotic treatment.

## Background

Endometrial polyps (EPs) is common benign gynecological protrusions which represent a localized hyperplastic overgrowth of stroma and endometrial glands; the prevalence of EPs ranges from 7.8 to 34.9% across different populations [1]. Previous studies have indicated that EPs is the result of hormonal dysfunction, although inflammatory factors such as chronic endometritis (CE) are also thought to play important roles in the development of EPs [2]. The prevalence of CE in premenopausal women with abnormal bleeding or reproductive failure on EPs was 28.7% [3].

CE is a persistent localized inflammatory condition of endometrium characterized by the presence of plasma cell infiltrate in the stroma [4, 5]. CE is usually asymptomatic or presents only with subtle symptoms such as abnormal uterine bleeding, pelvic pain, dyspareunia, and leucorrhea; which are very similar to the symptoms of EPs [6]. CE is associated with infertility, recurrent miscarriage and recurrent implantation failure [7, 8]. However, the spontaneous cure rate of CE was very low, 12.7% reported in a randomized clinical trial [9]. Many studies have shown that the cure rate of CE ranged widely from 27.9 to 92.3% with one course antibiotic because of different treatment regimens and different diagnostic creteria [10–13].

Dydrogesterone is a selective progesterone receptor agonist with excellent oral bioavailability and potent progestogenic activity, while having no androgenic, glucocorticoid or estrogenic activity, making it a good option for progestin therapy [14]. The safety of dydrogesterone has been confirmed in previous literature [15]. The transformation dose of dydrogesterone required for the secretory transformation of estrogenized human endometrium was 140 mg, leading to effective shedding of endometrium [16]. It can theoretically remove plasma cells and pathogenic bacteria in superficial layer. Our previous study had showed that addition of dydrogesterone was effective for the treatment of chronic endometritis with antibiotic treatment in premenopausal women [17]. Hysteroscopic resection has long been considered as the gold standard for the treatment of EPs [18]. Post hysteroscopic progesterone hormone therapy, dydrogesterone of 10 mg twice a day, from day 15 to day 24 of the menstrual cycle, had favorable clinical effect in treating EPs as it can effectively prevent the recurrence of EPs, relieve the level of hemoglobin and reduce endometrial thickness [19].

In the retrospective study we investigated whether addition of dydrogesterone with antibiotic treatment increases the cure rate of CE in premenopausal women who underwent hysteroscopic polypectomy.

## Methods

### Participants

This retrospective study was conducted from January 2016 to November 2020 in the Department of Obstetrics and Gynecology of the Affiliated Hospital of North Sichuan Medical College in Nanchong, China. Women diagnosed with CE by CD138 while underwent hysteroscopic polypectomy in proliferative phase were enrolled in this study. The study was approved by the Institutional Review Board of the Affiliated Hospital of North Sichuan Medical College. All patients signed the written informed consent form before participation in the study.

The inclusion criteria were EPs with CE diagnosed by CD138; agreement to undergo hysteroscopy, hysteroscopic resection, and endometrial biopsy; no contraindications to dydrogesterone or doxycycline; no severe systemic diseases. The exclusion criteria were history or presence of endometrial carcinoma; use of hormone replacement therapy or hormonal therapy in the preceding 3 months.

### Procedure

In the retrospective observational study, single antibiotic was given for the treatment of CE previously, and addition of dydrogesterone was used to prevent recurrence of EPs in women with CE with antibiotic treatment. The assignment of the medical intervention is not at the discretion of the investigator. From January 2016 to December 2018, women with CE who underwent hysteroscopic polypectomy were given single antibiotic for the treatment of CE, oral doxycycline 200 mg daily for 14 days. From January 2019 to November 2020, women with CE who underwent hysteroscopic polypectomy were given one course of dydrogesterone to prevent recurrence of EPs, oral dydrogesterone of 10 mg twice a day, from day 15 to day 24 of the menstrual cycle, except for the above antibiotic, oral doxycycline 200 mg daily for 14 days. In both groups, antibiotic was given once CE was diagnosed by CD138. A second look hysteroscopy was performed and endometrial biopsy sample was obtained after completion of therapy in the next proliferative phase to assess the response to treatment. Comparison of cure rates of CE with different treatment regimens was performed.

### Hysteroscopic polypectomy, hysteroscopy and endometrial biopsy

Hysteroscopic polypectomy was conducted in the proliferative phase under intravenous anesthesia using a bipolar resection system, containing a 3-mm 15° inside rigid hysteroscope and an 8.5-mm outside sheath (Olympus, Tokyo, Japan). Endometrial biopsies were performed to exclude endometrial lesions using a unelectrified plasma cutting ring away from the local polyps. Second look outpatient hysteroscopy was conducted in the proliferative phase without any anesthesia using a 3-mm 30° inside rigid hysteroscope and an 4.5-mm outside sheath (Olympus, Tokyo, Japan). Endometrial biopsies were obtained with the use of a metal curette, from the hysteroscopic features area, if not, from the upper uterine cavity blindly.

### Endometrial scratching

Endometrial scratching was performed in women with childbearing desire, using a unelectrified plasma cutting ring while hysteroscopic polypectomy. The scratching was performed once in each quadrant of the endometrium, away from the local EPs.

### Diagnosis of CE and immunohistochemistry

**Fig. 1:**
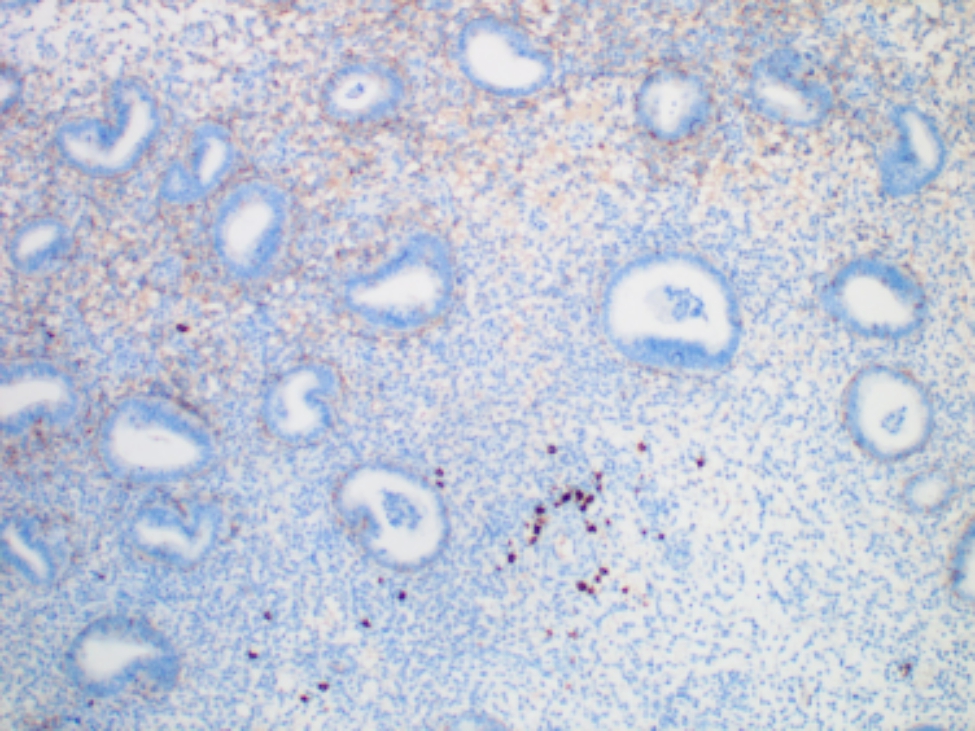
Diagnostic criteria of CE. CE was diagnosed by CD138 with one or more plasma cell identified per 10 high power fields. At least 50 high-power fields were examined per specimen

Currently, there is no consensus on the diagnostic criteria of CE. CD138 was the preferred immunohistochemical stain to identify plasma cells [3, 17, 20–22]. Pathologists have different views on the diagnosis of CE: how many CD 138^+^ cells/HPF [21]. However, CE decreased the pregnancy rate and the live birth rate when CE was diagnosed as the presence of ≥ 1 plasma cells in 10 high-power fields [22]. In the present study, according to the clinically relevant CE, CE was diagnosed by CD138 with one or more plasma cell identified per 10 high power fields (Fig. 1), as widely used in other studies [3, 17, 20–22]. At least 50 high-power fields were examined per specimen. Endometrial samples for routine histologic analysis and immunohistochemistry were conducted as previously described [20]. The anti-CD138 monoclonal antibody used in our study was MI15 Cell Marque (Fuzhou Maixin Biotechnology Co., Ltd., Fuzhou, China).

Slides immunostained for ER (estrogen receptor) and PR (progesterone receptor) were scored using the Allred Score [23, 24]. The clone of anti-ER and anti-PR monoclonal antibody used in our study were SP1 and SP2 (Fuzhou Maixin Biotechnology Co., Ltd.). ER and PR were classified as being positive if Allred score equal or more than 3.

### Statistical analyses

All analyses were conducted with the use of SPSS version 22.0 and a P value of < 0.05 was considered to be represent statistical significance. After analyzing the distribution of our data and confirming that age and body mass index (BMI) in the population were not normally distributed, we adopted a nonparametric method to analyze the age and BMI. Data are expressed as median (interquartile range) or percentage. The other intergroup differences were compared using Chi-squared test. Chi-square test was used to compare the conversion of CD138 between groups and subgroups. Logistic regression was carried out to investigate the factors associated with cure rate of CE.

## Results

### Comparison of general and clinical features of patients

**Fig. 2:**
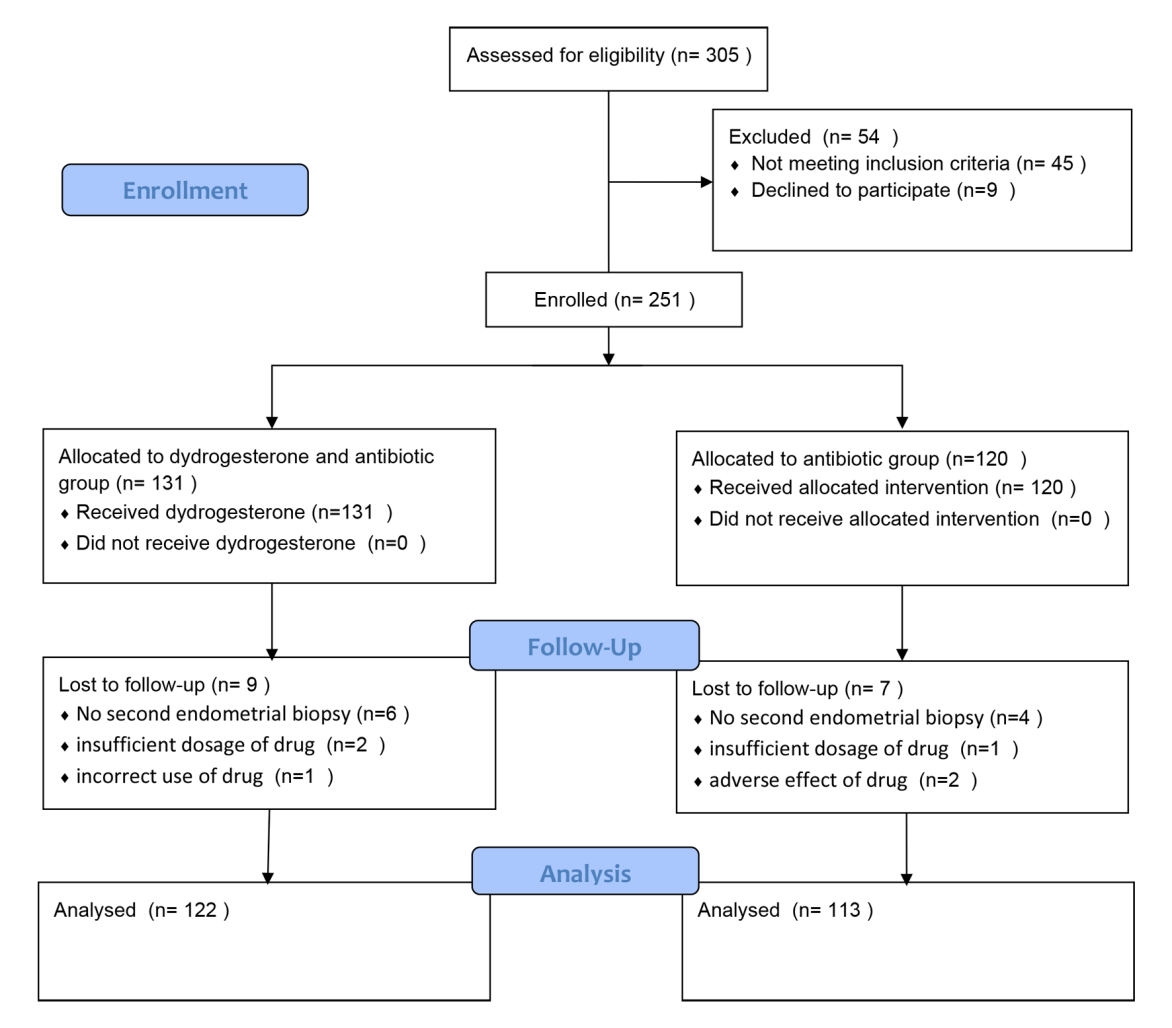
Flow diagram

During the 5-year study period, A total of 251 premenopausal women with CE who underwent hysteroscopic polypectomy were enrolled in this study. Sixteen cases were excluded for various reasons. Finally, 235 cases were enrolled in the statistical analysis, including 122 cases with dydrogesterone and doxycycline, and 113 cases with doxycycline alone (Fig. 2). The demographic details and clinical features of the two groups are shown in Table 1. The demographic details and clinical features of the two groups were not statistically significant (P > .05).

**Table 1:** Baseline demographic and clinical characteristics of the enrolled patients

**Characteristics**	**Dydrogesterone and antibiotic group (n = 122)**	**Antibiotic group (n = 113)**	***P value***
Age, y (median (Q3-Q1))	32 (6)	34 (8)	0.081
BMI, kg/m^2^ (median (Q3-Q1))	22.0 (3.7)	22.3 (4.6)	0.673
Polyp number, n (%)			0.714
One	35 (28.7%)	30 (26.5%)	
Two or more	87 (71.3%)	83 (73.5%)	
Endometrial scratching, n (%)			0.543
Yes	76 (62.3%)	66 (58.4%)	
No	46 (37.7%)	47 (41.6%)	
ER status, n (%)			0.061
Positive	104 (85.2%)	105 (92.9%)	
Negative	18 (14.8%)	8 (7.1%)	
PR status, n (%)			0.462
Positive	104 (85.2%)	100 (88.5%)	
Negative	18 (14.8%)	13 (11.5%)	

Note: BMI - body mass index; ER - estrogen receptor; PR - progesterone receptor

### Comparison of cure rate of CE between the two groups

**Fig. 3:**
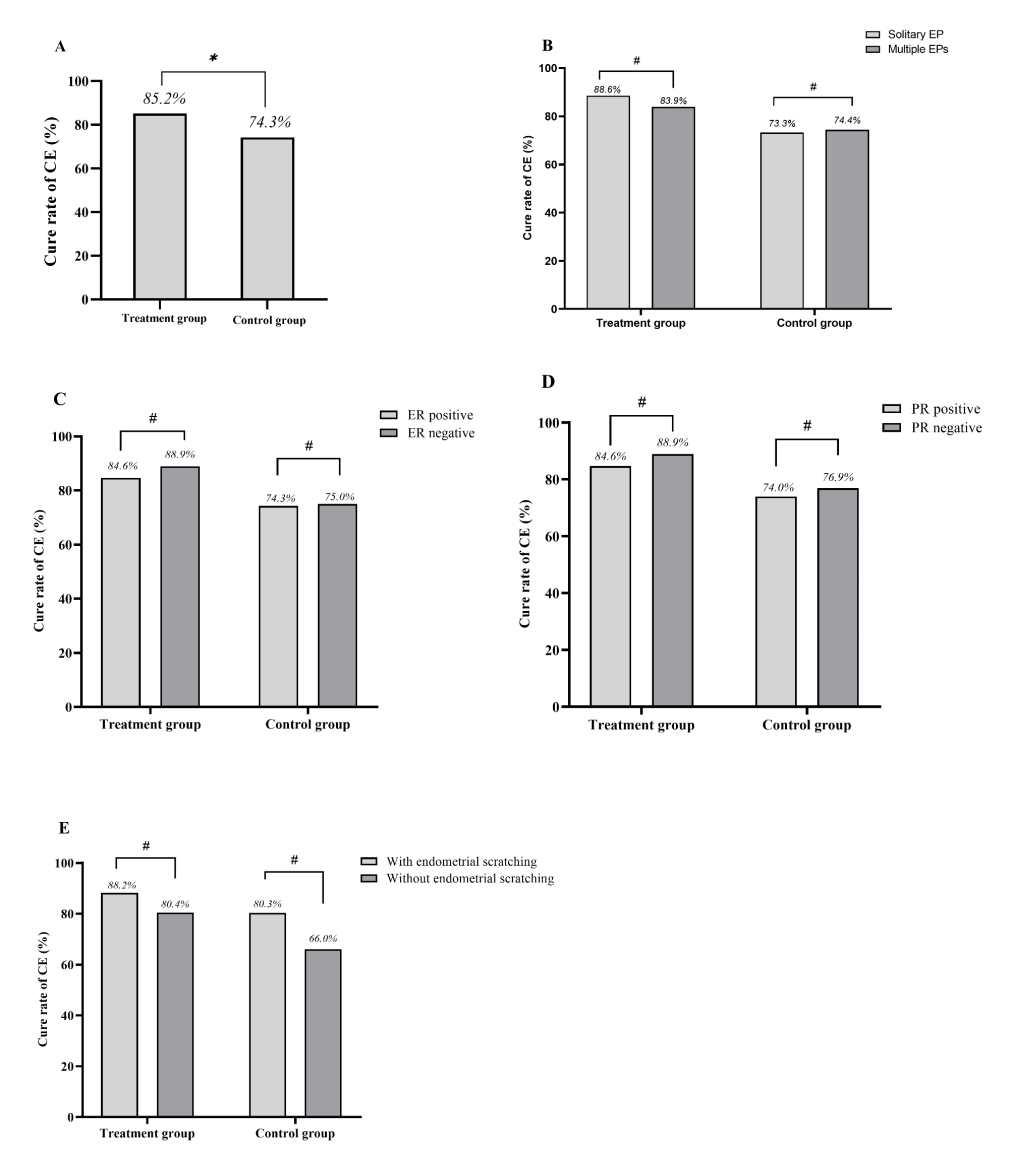
Comparison of the cure rate of CE between the treatment and control groups as well as subgroups in each group. (A) Comparison of the cure rate of CE between the treatment and control groups (*P < .05). (B) Comparison of the cure rate of CE among patients with different number of EPs in each group (#P > .05). C and D. Comparison of the cure rate of CE in according to ER and PR status of patients in each group (#P > .05 and #P > .05, respectively). E. Comparison of the cure rate of CE with or without endometrial scratching in each group (#P > .05)

The conversion of CD138 from positive to negative indicated the cure rate of CE. After treatment, the overall cure rate of CE in the population was 80.0% (188/235). The dydrogesterone and antibiotic group showed better effects regarding the cure rate of CE (Fig. 3, P < .05). The cure rate of CE was 85.2% (104/122) in the dydrogesterone and antibiotic combination group vs. 74.3% (84/113) in the single antibiotic group (Fig. 3, P = .037). Among patients with endometrial scratching, the cure rate of CE was 88.2% (67/76) in the dydrogesterone and antibiotic combination group vs. 80.3% (53/66) in the single antibiotic group (P = .197), while without endometrial scratching, the cure rate of CE was 80.4% (37/46) in the dydrogesterone and antibiotic combination group vs. 66.0% (31/47) in the single antibiotic group (P = .115).

### Comparison of cure rate of CE between subgroups in the treatment group

In the treatment group, the cure rates of CE were 83.9% (73/87) in women with solitary EP and 88.6% (31/35) in women with multiple EPs, respectively (Fig. 3, P = .586). This may suggest that the effect of dydrogesterone was not associated with the number of EPs. The cure rates of CE were 84.6% (88/104) in women with ER positive and 88.9% (16/18) in women with ER negative, respectively (Fig. 3). The cure rates of CE were 84.6% (88/104) in women with PR positive and 88.9% (16/18) in women with PR negative, respectively (Fig. 3). The influence of status of ER or PR was undefined because of high expression of ER and PR in women with CE and EPs.

### Factors associated with cure rate of chronic endometritis

Multivariate analysis confirmed that addition of dydrogesterone was a beneficial factor for cure rate increase with antibiotic treatment (Table 2; OR, 2.10 [95% CI, 1.06–4.15]; P = .032). In addition, endometrial scratching also contributed to cure rate increase with antibiotic treatment (Table 2; OR, 2.24 [95% CI, 1.14–4.39]; P = .019). The cure rates of CE with and without endometrial scratching were 84.5% (120/142) and 71.8% (68/93), respectively (P = .033). endometrial scratching was effective for the treatment of CE. However, in the treatment group, the cure rates of CE with and without endometrial scratching were 88.2% (67/76) and 80.4% (37/46), respectively (Fig. 3, P = .244). This finding may indicate that endometrial scratching did not function in women using dydrogesterone for CE. Conversely, multivariate analysis showed that the cure rate of CE was not affected by age, BMI, number of EPs, ER status and PR status.

**Table 2:** Multivariate logistic regression analysis of factors associated with cure rate of chronic endometritis

**Variables**	**Cure rate of CE** **OR (95% CI)**	***P value***
Treatment (dydrogesterone and antibiotic)	2.10 (1.06–4.15)	0.032
Endometrial scratching	2.24 (1.14–4.39)	0.019

## Discussion

In this retrospective trial of 235 participants with CE who underwent hysteroscopic polypectomy, combined administration of dydrogesterone and antibiotic had a higher cure rate of CE compared with the treatment of antibiotic alone. The finding supported a beneficial role of co-treatment with dydrogesterone and antibiotic in premenopausal patients with CE who underwent hysteroscopic polypectomy.

Many studies have shown that impaired inflammatory state of the endometrium (IISE) is the main cause of most intrauterine diseases, such as EPs, unexplained infertility and endometrial cancer [25]. Unlike CE, IISE contains infectious and non-infectious etiology [25]. Dydrogesterone is effective in the treatment of EPs in premenopausal women [26]. Furthermore, it can effectively prevent the recurrence of EPs after hysteroscopic polypectomy [19]. To our knowledge, this is the first study to assess the treatment of CE after hysteroscopic polypectomy with combination of dydrogesterone and antibiotic.

In this study, women with combination of dydrogesterone and antibiotic had a higher cure rate of CE in one course compared with women with antibiotic alone. The overall cure rate of CE in premenopausal women was 80.0%. The cure rate of CE with single doxycycline was 74.3%, which is consistent with previous studies [10, 12, 17]. With the use of dydrogesterone, the cure rate of CE reached to an elevated level of 85.2%. Multivariate analysis confirmed that dydrogesterone was a beneficial factor for cure rate increase with antibiotic treatment (OR, 2.10 [95% CI, 1.06–4.15]; P = .032). The potential mechanisms underlying the beneficial effect of dydrogesterone is currently unknown. One of the possible explanation is that dydrogesterone application can remove plasma cells and pathogenic bacteria in the superficial layer [16]. Synergistic effect occurred with the effect of dydrogesterone to reduce the severity of CE. In addition, dydrogesterone may improve the local immune status within the endometrium [14]. Furthermore, progesterone has been found to function in cell apoptosis of endometrium [27–29].

It remains unclear if endometrial scratching improves the chance of pregnancy and, if so, for whom [30]. The procedure is painful, with patients reporting pain scores of 3–7 out of 10, causes bleeding, and carries a risk of infection, it entails the inconvenience of attending an additional clinic and additional charge [31]. In the current study, endometrial scratching was conducted while hysteroscopic polypectomy under intravenous anesthesia, it can ovoid the above hazard factors. Multivariate analysis confirmed that endometrial scratching was a beneficial factor for cure rate increase with antibiotic treatment (OR, 2.24 [95% CI, 1.14–4.39]; P = .019). This may be the explanation that endometrial scratching improves the chance of pregnancy by curing the concealed CE. However, with the use of dydrogesterone, the cure rates of CE with and without endometrial scratching were 88.2% (67/76) and 80.4% (37/46), respectively (P = .244). There was no additive function with dydrogesterone and endometrial scratching for the treatment of CE.

There are limitations in this study which should be considered. For example, in the retrospective review, we can not cover plentiful enough impact factors for cure rate of CE, concealed factors need to be explored. Furthermore, only women with CE on EPs were enrolled, women with CE on other diseases are needed for widely application. Large, prospective studies will be necessary to confirm the beneficial role of co-treatment with dydrogesterone and antibiotic in patients with CE and the potential mechanisms.

## Conclusion

In conclusion, combination of dydrogesterone and antibiotic was more effective for CE than antibiotic alone in premenopausal women after hysteroscopic polypectomy. Synergistic effect occurred with the effect of dydrogesterone to reduce the severity of CE. With the same reason, endometrial scratching also contributed to the cure rate increase with antibiotic treatment. However, there was no duplicate effect.

## Data Availability

The datasets used and/or analysed during the current study are available from the corresponding author on reasonable request.

## Abbreviations

Eps: endometrial polyps
CE: chronic endometritis
ER: estrogen receptor
PR: progesterone receptor
BMI: body mass index
IISE: impaired inflammatory state of the endometrium
